# The African Immigrant Memory Loss Project: A University-Community Partnership

**DOI:** 10.1093/geroni/igaa057.1064

**Published:** 2020-12-16

**Authors:** Manka Nkimbeng, Hayley McCarron, Gabriela Bustamante, Wynfred Russell, Tetyana Shippee, Joseph Gaugler

**Affiliations:** 1 Johns Hopkins University school of Nursing, Minneapolis, Minnesota, United States; 2 University of Minnesota, Minneapolis, Minnesota, United States; 3 African Career, Education & Resource, Inc., Brooklyn Park, Minnesota, United States

## Abstract

The few studies on dementia prevalence in immigrant communities show that immigrants from diverse racial/ethnic backgrounds have a higher prevalence of dementia compared with their U.S.-born counterparts. However, this body of work is small, resulting in a lack of reliable estimates of dementia prevalence among African immigrants. The AIMLP is a partnership between the African Career, Education, and Resources, Inc. (ACER) and the Families and Long-Term Care Projects (FLTC) of the University of Minnesota School of Public Health. Guided by an advisory board, the goal of this project is to develop culturally informed instruments, and use these to collect data to identify dementia care needs, knowledge, and resources in the African immigrant community. Study implementation started in August 2019, five advisory board meetings have been convened and 2 pilot focus groups have occurred. Twelve individuals participated in the focus groups. The majority (90%) were from Liberia and 60% were over the age of 55. Two participants currently care for a family member with dementia. Preliminary findings reveal a great need for education on dementia, and general lack of awareness on management, and limited access to services/supports. Focus groups will be finalized in March and the study survey will be developed and administered in the summer. These survey findings will be available and presented at the conference in November 2020. This is the first project to identify the extent of dementia care needs and resources among African immigrants; which will inform interventions for this population.

